# The complete chloroplast genome of the Tibetan medicinal plant *Rhodiola kirilowii*

**DOI:** 10.1080/23802359.2020.1861561

**Published:** 2021-01-21

**Authors:** Guoying Zhang, Yarong Liu

**Affiliations:** Qinghai Provincial Key Laboratory of Modernization of Traditional Chinese and Tibetan Medicine, Qinghai Provincial Drug Inspection and Testing Institute, Xining, China

**Keywords:** *Rhodiola kirilowii*, chloroplast genome, phylogenetic analysis

## Abstract

*Rhodiola kirilowii* is a widely used Tibetan medicine. Here, we report the complete sequence of the chloroplast genome of *R. kirilowii.* The genome was 150,896 bp in length with 131 genes comprising 85 protein-coding genes, 37 tRNA genes, 8 rRNA genes and 1 pseudogene, with 20 of them occurring in double copies. Phylogenomic analysis suggested that *R. kirilowii* forms a clade with *R. rosea*, *R. yunnanensis*, *R. fastigiata* and *R. crenulata* in *Rhodiola* genus.

*Rhodiola kirilowii* is a herbaceous perennial belonging to the family Crassulaceae (Fu and Ohba 2001). *Rhodiola kirilowii* is an important Tibetan medicine, from which various distinct groups of chemical compounds (phenylethanol derivatives) have been isolated (Yang 1991; Grech-Baran et al. 2015). It was demonstrated that *R. kirilowii* could be effective in increasing immunity, stimulating nervous system, eliminating fatigue and preventing high-altitude sickness (Panossian et al. 2010). Here, we report the completed chloroplast genome of *R. kirilowii* and investigate its phylogenetic relationship within Crassulaceae, which may be conductive to a deeper understanding of this particular species.

*Rhodiola kirilowii* was collected from Baoku Forestry Farm (Qinghai, China；N 37°07′06″, E 101°34′42″). The voucher specimen (Chi202001) was stored in Qinghai-Tibetan Plateau Museum of Biology (HNWP). Total genomic DNA was obtained from silica-gel dried leaves of *R. kirilowii* by modified CTAB method (Doyle 1987). Sequencing was performed on the Illumina HiSeq2500 platform (San Diego, CA, USA). The chloroplast genome was assembled by SPAdes (Bankevich et al. 2012), annotated by CpGAVAS (Liu et al. 2012).

A typical quadripartite structure with a total length of 150,896 bp was found in the *R. kirilowii* chloroplast genome. The large single-copy region (LSC, 82,233 bp) and the small single-copy region (SSC, 16,992 bp) were separated by two inverted repeats (IR, 25,834 bp). Totally 131 genes were predicted in the genome, including 85 protein-coding genes, 37 tRNA genes, 8 rRNA genes and 1 pseudogene. 9 protein-coding genes (ndhB, rpl2, rpl23, rps7, rps12, rps19, ycf1, and ycf2), 4 rRNA genes (4.5S, 5S, 16S, and 23S rRNA) and 7 tRNA genes (trnA-UGC, trnI-CAU, trnI-GAU, trnL-CAA, trnN-GUU, trnR-ACG, and trnV-GAC) duplicated in the IR regions.

Phylogenetic analysis was performed among *R. kirilowii* and 24 species in Crassulaceae with *Saxifraga sinomontana* as the outgroup. The whole plastome sequences were used for the phylogenetic analysis. Phylogenetic analysis was conducted on the PhyloSuite platform (Zhang et al. 2020). All of the sequences were aligned using MAFFT. ModelFinder were used to select the best model for phylogenetic analyses. The phylogenetic trees were constructed using IQ-TREE with 1000 bootstraps. All the generated trees were modified by Interactive Tree Of Life (iTOL, http://itol.embl.de/) (Figure 1). The phylogenetic tree showed that *R. kirilowii* forms a clade with *R. rosea*, *R. yunnanensis*, *R. fastigiata* and *R.crenulata.*

**Figure 1.**
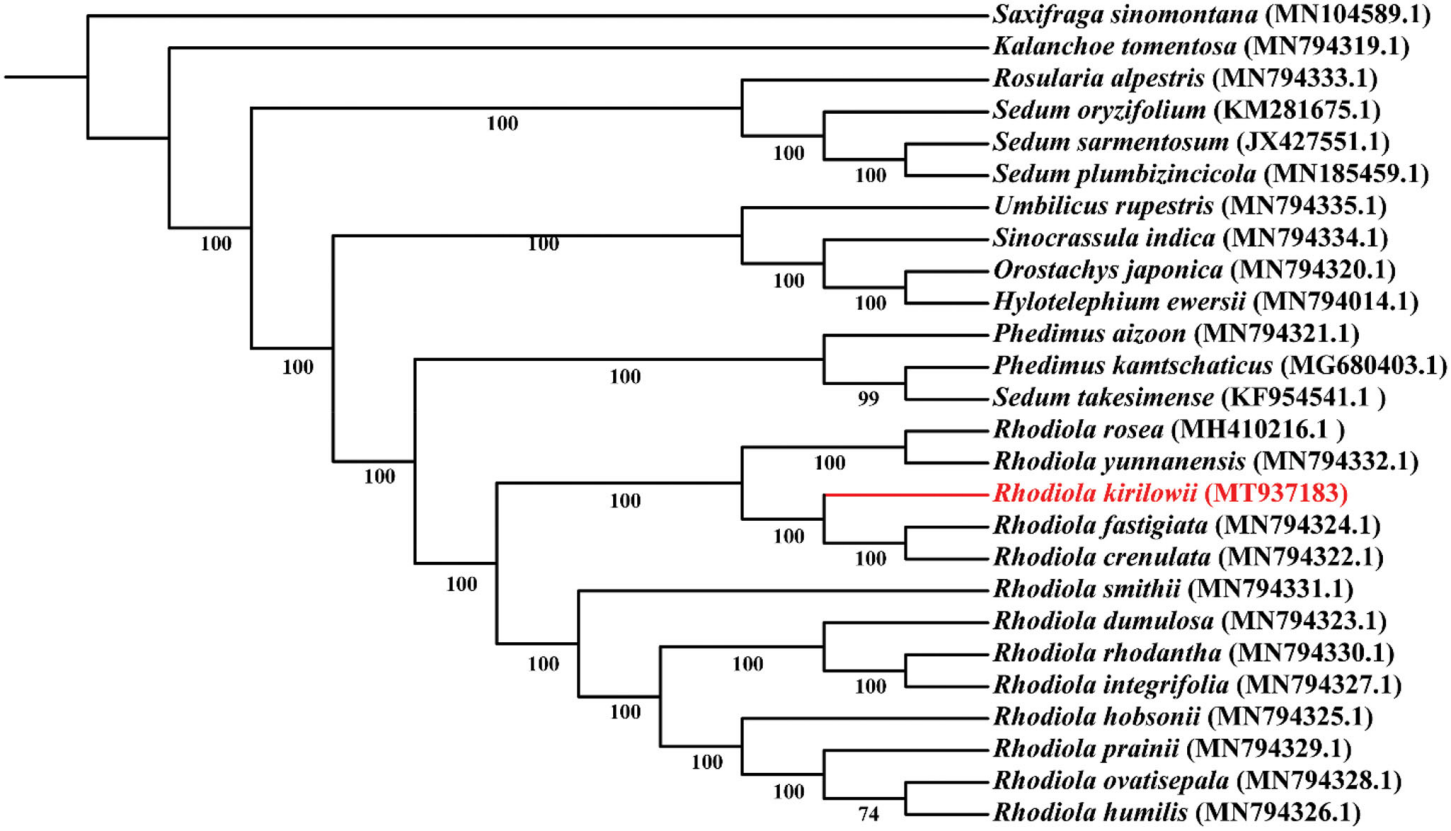
IQ-TREE based on 26 complete chloroplast genome sequences. The number on each node indicates the bootstrap value.

## Data Availability

The chloroplast genome and raw sequencing data in this study are available in GenBank (https://www.ncbi.nlm.nih.gov/) under the accession numbers of MT937183 and SRR12991102.
